# 

**Published:** 1842-09-17

**Authors:** 


### MEDICAL EXAMINER,

EDITED BY

*J. B. BIDDLE, M.D. & W. W. GERHARD, M.D.*

VOL. I.] SEPTEMBER 17, 1842. [No. 38.

